# Supplementary material for: Nanoshaped Cerium Oxide with Nickel as a Non-Noble Metal Catalyst for CO2 Thermochemical Reactions
Source: Molecules. 2023 Mar 24;28(7):2926. doi: 10.3390/molecules28072926 (PMC10095831; doi:10.3390/molecules28072926)
Supplement: Supplementary file 1 [file molecules-28-02926-s001.zip › molecules-2153718-supplementary.pdf]

molecules

Supporting information

Nanoshaped Cerium Oxide with Nickel as a Non-Noble Metal Catalyst for CO_2 Thermochemical Reactions

Content:

Figure S1. Adsorption - desorption isotherms of nitrogen for cerium dioxide shapes (a), and pore size distribution for cerium dioxide shapes (b). 2

Figure S2. N_2 adsorption-desorption isotherms (a) and pore size distribution (b). 3

Figure S3. SEM pictures of the Ni-CeO_2 rods catalysts: (a) 0.5%-Ni-CeO_2 rods; (b) 1%-Ni-CeO_2 rods; (c) 2%-Ni-CeO_2 rods; (d) 5%-Ni-CeO_2 rods; (e) 10%-Ni-CeO_2 rods. 5

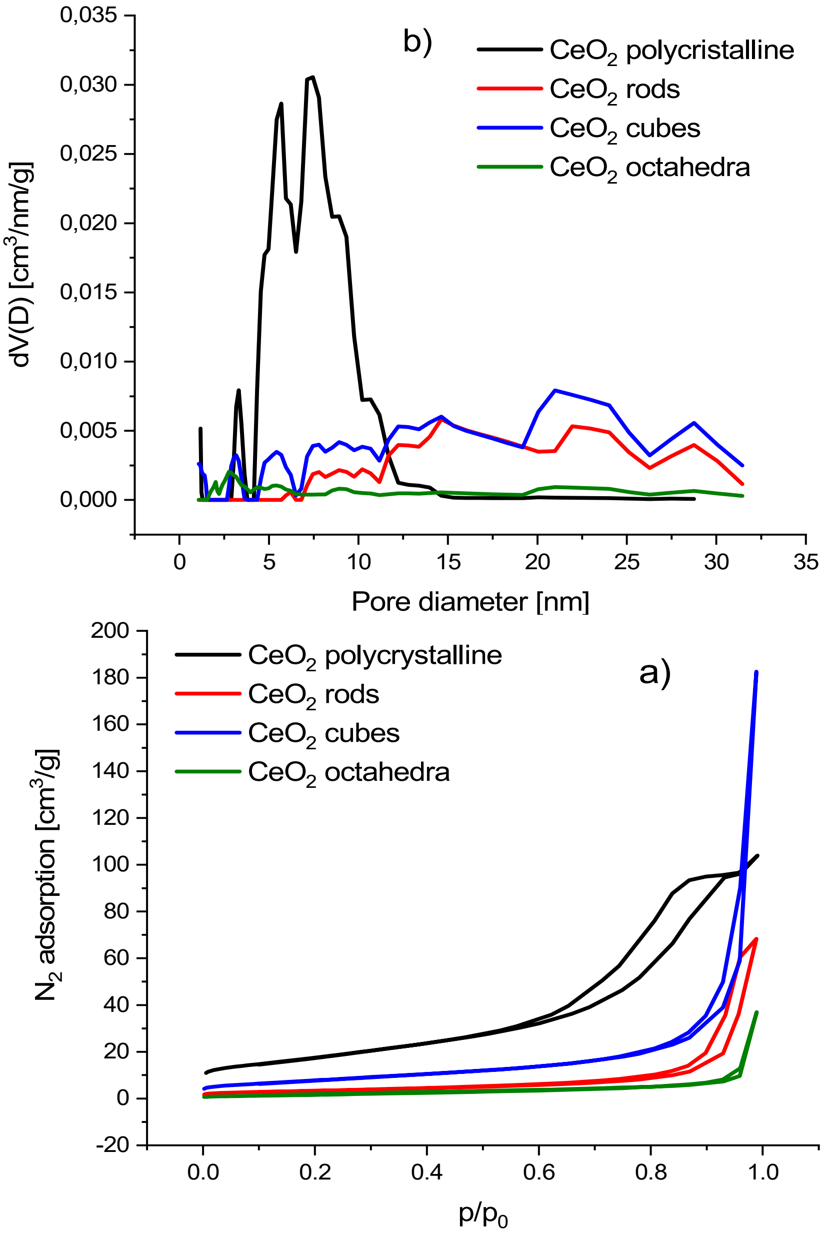

Figure S1. Adsorption - desorption isotherms of nitrogen for cerium dioxide shapes (**a**), and pore size distribution for cerium dioxide shapes (**b**).

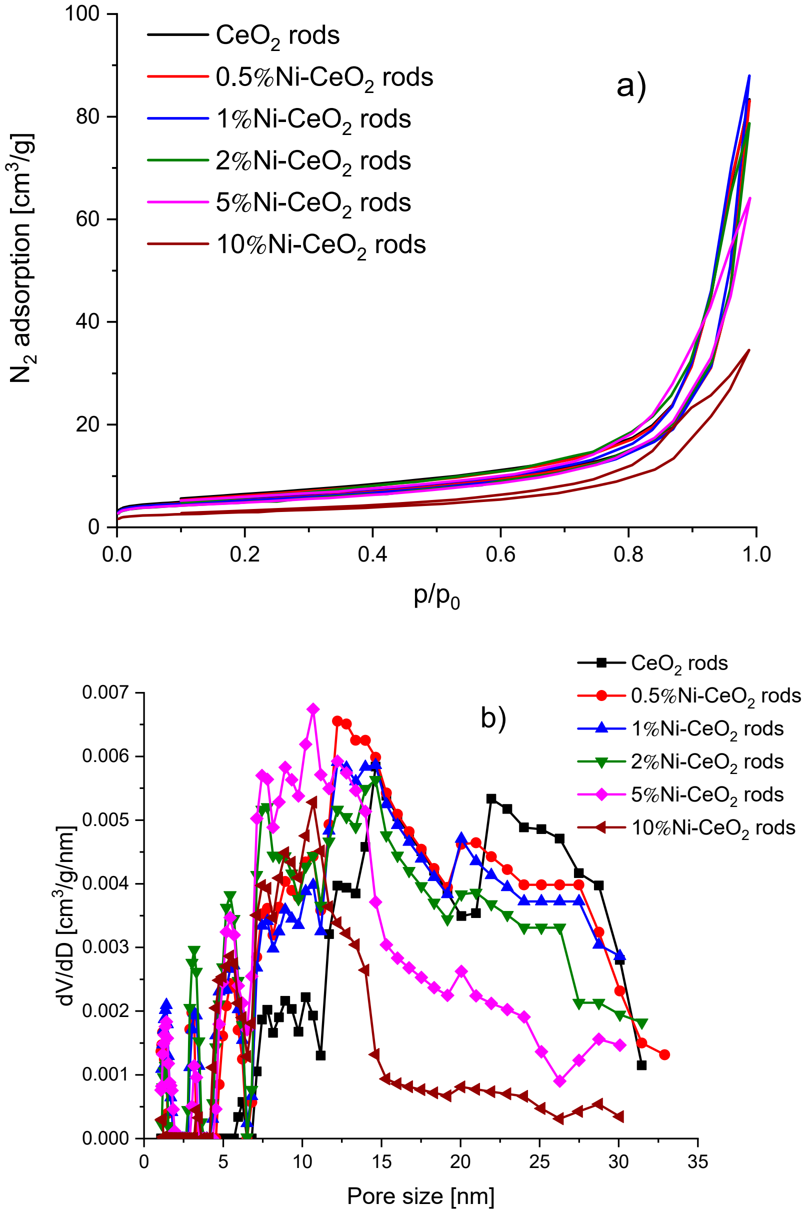

Figure S2. N_2 adsorption-desorption isotherms (**a**) and pore size distribution (**b**).

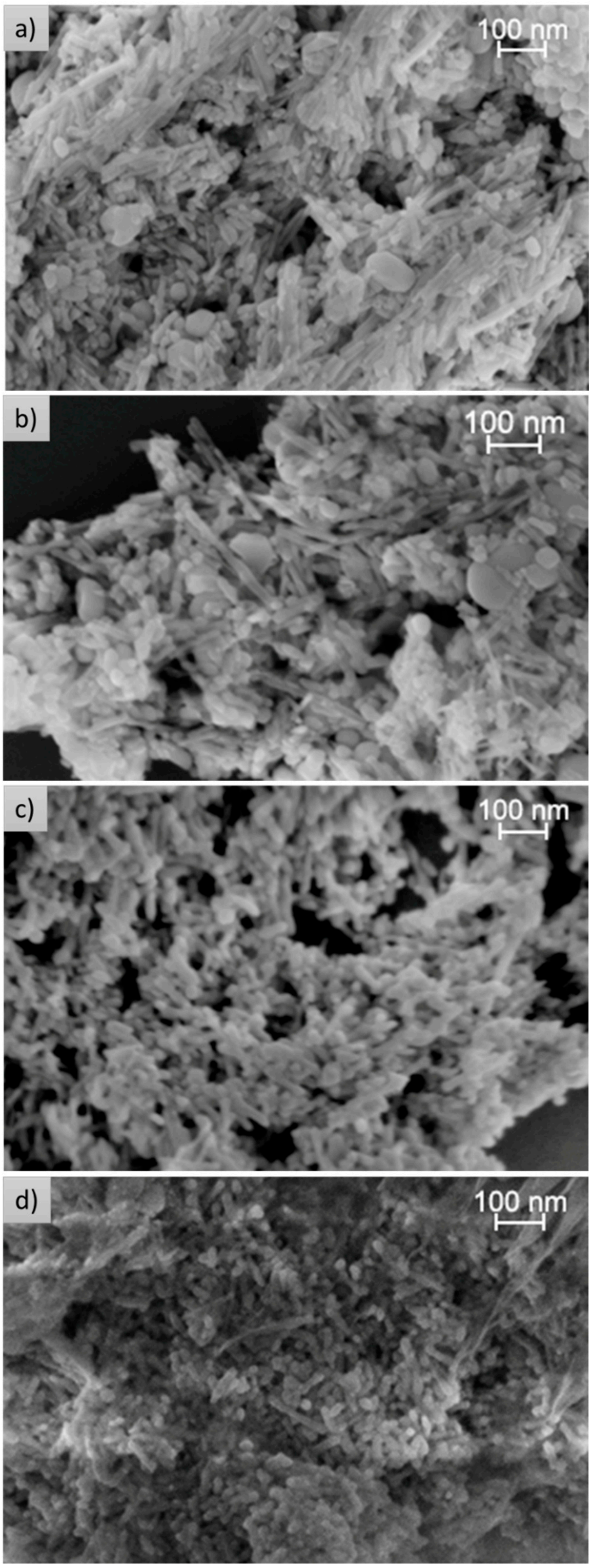
a)
100 nm
b)
100 nm
c)
100 nm
d)
100 nm

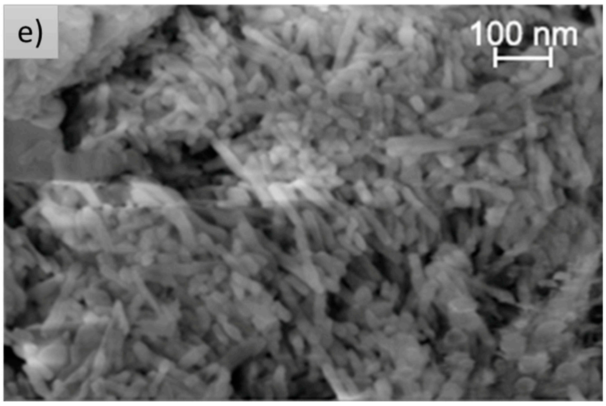

Figure S3. SEM pictures of the Ni-CeO_2 rods catalysts: (**a**) 0.5%-Ni-CeO_2 rods; (**b**) 1%-Ni-CeO_2 rods; (**c**) 2%-Ni-CeO_2 rods; (**d**) 5%-Ni-CeO_2 rods; (**e**) 10%-Ni-CeO_2 rods.
